# The Hospital Nursing Supplement

**Published:** 1893-04-22

**Authors:** 


					The Hospital, Aran, 12,1393. Extm SnpphmeM..
**
Hospital" Hurstttg fttttror.
Being ?hb Extra Nursing Supplement of "The Hospital" Newspaper.
[Contributions for thia Supplement should be addressed to the Editor. The Hospital, 423, Strand, London, W.O., and should have the wor.i
Nnrsmtr" plainly written in left-hand top corner of the envelope.]
IRews from tbe IFlursinc} THUorlD.
THE POPLAR HOSPITAL FOR ACCIDENTS.
Under the patronage of H.R.H. Princess Louise,
Marchioness of Lome, and H.R.H. Princess Mary of
Teck, a matinee is to be given at the Trafalgar Square
Theatre on May 2nd in aid of the funds of the Poplar
Hospital for Accidents. The programme is excellent
and varied, and cannot fail to attract a large audience
and to considerably benefit tbe excellent institution for
?which it has been so kindly planned.
THE INVALID CHILDREN'S AID ASSOCIATION.
Another matinee is to be given at the Trafalgar
Square Theatre on April 25th, under the patronage of
H.R.H. Princess Mary of Teck and her daughter
Princess Mary Victoria. The latter is the patron of
tbe I.C.A.A., of which the Earl of Aberdeen is presi-
dent. This useful association urgently needs additional
funds to carry on and to increase the work it under-
takes amongst the invalid and crippled child ren of the
London poor.
A POPULAR HOME SISTER.
Members of the Nurses' Co operation in New Caven-
dish Street are all regretting the departure of Miss
Leigh, the Home Sister, who has done bo much for their
comfort during more than two years. Her resignation
was accepted with great regret by the committee, who
are fully conscious of the able assistance which Miss
Leigh has rendered to the Lady Superintendent ever
since the Nurses' Co-operatiou was first started. The
sincere good wishes of all who know her will accompany
Sister Leigh to her new work.
TRAINED TO WORK.
Women are able to compete with men in numerous
ways, and with a fair amount of success at present.
Unluckily, neither girls nor their parents seem, how-
ever, to understand that for all kinds of work training
is needful. The length of this must naturally depend
upon the kind of employment aimed at. It is no un-
usual thing to hear the friends of a young probationer
speaking with great contempt of the hospital system
which rules that " quite a ridiculously small salary " is
all she will get for her first year's work. " Such hard
work, too; dear Maria ought to be well paid for it!"
But these undiscerning friends do not realize that Maria
is simply serving an apprenticeship that she may
eventually rank us a duly qualified, certificated nurse.
It would be better for many a probationer if her
friends knew what a Bhort experience generally proves
to herself that a full and faithfully served apprentice-
ship to the art of nursing is the only method by which
she can adequately fit herself to take any good posi-
tion in the profession to which sbe aspires to belong.
EASE INKILLNESS.
Exactly how many people come up to London
every year to undergo small or large operations it is
well-nigh impossible to estimate. Comparatively few
of the number have friends who can olfer sufficient
accommodation for patient and nurses, therefore a suite
of apartments must be engaged, and the many dis-
comforts of furnished lodgings encountered, unless
refuge is sought in a regular "Nursing Home." By
residents in town the Nursing Home is often found to
he equally valuable. Skilled nursing is there facili-
tated by so many appliances, as the accessories are
naturally chosen with intent to lighten the burden of
sickness by all possible means. In counting the cost
of residence in a Nursing Home, it is well to remember
that the terms not only cover the various expenses of
nursing and maintenance, but also many unenumerated
comforts of whose existence the unprofessional patient
has hitherto been ignorant. Human nature easily
reconciles itself to increased luxury, and people in good
Home Hospitals soon begin to pity persons less per-
fectly tended. A charming new establishment of
this description has just been opened at 11, Duchess
Street, Portland Place, by three ladies. Being fully
trained and certificated hospital nurses, they are prepared
and qualified to undertake medical, surgical, and
accouchement cases. Their pretty and convenient
house well repays a visit, and inclines the healthy
guest to say, "It would be no great hardship to ber ill
in such a home as this."
A GOOD REPORT.
Beaintree and Bocking Cottage Hospital shows an
excellent balance-sheet for 1892, owing to the increase
in donations and subscriptions. The house to house
canvass made by some ladies and gentlemen proved
most successful in adding to the subscription list.
HOPE DEFERRED.
The trained nurses connected with the institute at
Bedford must have almost given up hoping for the
increase of space and greater comfort once talked of
for them. It is ten months since the bazaar was held,
by which a sum of ?600 was raised "to benefit the in-
stitute." Apparently the governors cannot make up their
minds whether to enlarge the one existing or to erect
a new building. No doubt the decision presents certain
difficulties, but it is disheartening to busy workers to
find, after a lapse of so many months, the money in-
tended to benefit the Home is still lying idle.
A BAD EXAMPLE.
It is the fashion in some institutions to look upon
illness as criminal when any members of the staff of
workers indulge in it. The idea may sound preposter-
ous outside hospital walls, but dwellers within the pre-
cincts of some of these places know quite well that they
are not expected to be ill, and will therefore get scant
pity if they indulge in this luxury. However, it is
somewhat exceptional for even a Board of Guardians to
carry economy in the nursing department to quite such
an extent as the gentlemen at Horsham have done. A
nurse, supplied to them by the Workhouse Nursing
Association, was unlucky enough to contract small-pox,
and this is said to have cost the ratepayers ?12. There-
fore Colonel Lyon proposed, at the last board meeting,
xxxii THE HOSPITAL NURSING SUPPLEMENT. apkil 22,1893-
that the annual donation of ?2 23., due to the W.N. A.,
should not be paid this year. It seems to an unpre-
judiced reader that, after pitying the nurse, a little
condolence is also due to the association, which loses
for the moment, not only the services of a valuable
worker, but a'so one of the donations of which it stands
urgently in need. The aims of this society are so ex-
cellent that it deserves all the support possible, and the
action of the Horsham Board should be considered a
warning, certainly not an example, for imitation.
MANX DISTRICT NURSING.
The present state of the fund which nominally sup-
ports the Town Nurse at Douglas, Isle of Man, may be
described as unsatisfactory. Two years ago a meeting
was held, at which ministers of all denominations were
present, and apparently they cordially supported the
movement; but the hopes thus raised have not been
realised, for the nurse has worked for nearly eighteen
months, and " not one year's salary has been received
as yet," writes the Hon. Treasurer.
KILMARNOCK.
An additional nurse is needed for district work by
the Association at Kilmarnock, and we think it will
not be long ere sufficient funds are raised to justify
her appointment. The appreciation of the nurses and
the sympathetic interest evinced in their work at the
annual meeting gave every promise of increased sup-
port.
GOOD MANAGEMENT AT PERTH.
The present position of the Perth Sick Poor Nursing
Society is a most satisfactory one in all ways, and very
general interest is now shown in the work done by it.
The society aims at the propagation of a knowledge of
hygiene and sanitation, as well as the alleviation of
immediate suffering. During last year affiliation with
the Queen Yictoria Jubilee Institute for Nurses was
completed, and the Superintendent now holds the
diploma of a "Queen's Nurse." The Ladies' Com-
mittee show their interest in the society in many prac-
tical ways, such as providing a regular supply of soup
for the sick poor, &c., and other comforts.
ONE NURSE ONLY.
Doubtless most of our readers believe that the
necessity for increasing the nursing staffs of workhouse
infirmaries is not only getting generally known, but
universally acknowledged. Unfortunately, instances
constantly come to our notice of Boards of Guar-
dians considering one woman, whether trained or un-
trained. quite competent to attend to eighty or one
hundred sick persona, some perhaps hopelessly ill, if not
actually dying. It is painful to believe in the existence
of such callous indifference to the sufferings of the sick
poor. Recently the Coleraine Guardians advertised
for a nurse and an assistant nurse at decidedly mode-
rate salaries, viz., ?25 per annum, for the superior
appointment. They received applications, and the Local
Government Board sanctioned their choice of Mrs.
Mary Ellis, after which Mr. Hezlett gave it as his
opini in that no assistant nurse was required. The
Chairman reminded the gentlemen that they had ad-
vertised for one, which appears sufficient evidence of
the existing need. However, Mr. Hezlett's economical
idta was supported by one or two other members, but
the report says "the matter then dropped," which
leaves both no and our readers to form our own conclu"
sions as to the likelihood of the Coleraine "Workhouse
nurse being permitted the aid of " an assistant nurse."
THE MYSTERIES OF RED TAPE.
The officers of the Indian Army who are entitled to
passage money are allowed to draw the sum due to
them, whether they proceed to England or to one of the
Colonies. The same privilege was granted to members
of the Indian Parsing Service, on the understanding,
of course, that the cost should not exceed that of the
journey to England. For some unknown reason, how-
ever, the application of Miss G. M. Lickfold for the
regulated amount of passage-money to take her to
New Zealand has been refused by Government. As
Miss Lickfold, who has been Acting Superintendent at
Mhow, has completed her five years' term of service, it
is difficult for outsiders to fathom the reason for the
action of the authorities in this matter.
RELIEF STATIONS AT CHICAGO.
A very complete system of "relief stations," or
temporary hospitals, has been organised throughout
the grounds at the Chicago Exhibition. The cost of
establishment and maintenance will be shared between
the leading American hospitals and the various manu-
facturers of institutional appliances and fittings.
UNWELCOME VARIETY.
Another board of guardians finds itself face to face
with difficulties in the nursing department of its work-
house. The nurses at Cardiff are constantly resigning.
The Chairman inquired at a recent board meeting how
many nurses had left in six weeks, and the answer was
" Several." Evidently these incessant changes have
become so inconvenient that the guardians realise that
something must be done to put a stop to them.
Everything depends now on what the " something " is
The gentlemen assembled at the meeting agreed that
the " repeated resignations " of the nurses ought to be
prevented, and they referred the whole matter to the
consideration of the Hospital Committee. We sincerely
trust the latter possess sufficient knowledge on the
subject of skilled nursing to judge fairly of the rights
and wrongs of these transitory workers. We sympa-
thise with the sick inmates, and hope the difficulty of
securing pi*operly trained permanent nurses for them
may shortly be satisfactorily solved.
A PROSPEROUS IRISH HOSPITAL-
The little Monkstown Hospital is at present in a
position which many larger institutions may well
envy. The new buildings and their fittings are free
from debt, and the increased accommodation has en-
abled the medical staff to comply with the demands for
admission, although these have been unusually numer-
ous We hope Monkstown Hospital will be able to
Bubmit equally good reports to future subscribers, and
that these latter will steadily add to their list.
SHORT ITEMS.
The nurses at the Mater Misericordia Hospital,
Dublin, are favoured with three lectures every week
from members of the medical staff.?At the annual
meeting of the North London Nursing Association, a
vote of thanks was proposed to the superintendents
and nurses, "for the devotion they have shown in
carrying out their work amongst the sick poor."
THE DISABLED NURSE.
We have pleasure in acknowledging 4s. Gd. from
S. 0., making total ?12 14s. 6d. Of this, ?5 7s._6d.
has been handed to our invalid sister, who writes
gratefully of the acceptable help rendered by our
readers.
April 22, 1893. THE HOSPITAL NURSING SUPPLEMENT. xxxiii
Sanitation for IRurses*
By a Sanitary Inspector.
II.?notification in foreign countries.
Ifow that English nurses are to be found in nearly every
country on the face of the earth we cannot confine our
attention to sanitary law in England only, but must also
consider it in at least the principal countries in Europe.
Hence we propose to pass in rapid review the laws
regulating sanitary matters in France, Germany, Belgium,
Austria, &c.
Beginning with France we find organised sanitary regula-
tions in a somewhat backward state. There is here no
sanitary law at all resembling our own Health Acts, and
without any such guidance matters must be more or less
haphazard. The supreme control of sanitation in all its
branches has since 1889 been in the hands of the Minister of
the Interior, and immediately under him works the Comte
Consulatif d'Hygiene Publique. The Prefect of Police in
each district seems to have the chief authority. He has
no special training in or knowledge of sanitary
matters, but nevertheless he it is who has to decide whether
the medical officer shall or shall not make the necessary
sanitary investigations. The Prefect nominates what is
called a Health Committee for his district, which he can
consult or not at his discretion ; hence we are not surprised to
hear that in Eome places the committee has not met for two
or three years. It is obvious that the Prefect reigns
supreme in matters sanitary. In France, moreover, notifica-
tion of infectious dieeases is by no means compulsory, with
the single exception of lodging-houses. It is true there is
some semblance of the right thing in bo far as all doctors
are provided by the authorities with proper certificates to
311 up, in cases of infectious disease, but naturally where
their duty is not obligatory, it is not very likely to be per-
formed except by a few conscientious persons, and as a result
no complete reliable report of epidemics can be obtained.
Quite recently, however, the Paris Board of Health has
directed all doctors to report to the Commissioners of Police
every case of Influenza that comes under their notice. So
perhaps this step in the right direction may be the begin-
ning of better things.
At the present time, even in Paris, there are no special
hospitals for infectious diseases, though the advisability of
providing such has lately been recognized, so we may hope
soon to see the defect remedied. Small-pox iB only admitted
into two of the general hospitals of Paris, which have the
advantage of separate buildings in which it can be isolated.
?Other infectious diseases are admitted into general hospitals
and isolated as thoroughly as the accommodation will
permit; but judging by statistics, isolation does not appear
*0 be very successfully carried out, as infectious diseases are
very prevalent, and the mortality from them relatively
higher than in those countries where isolation is thorough.
When infectious disease breaks out, the municipal
authorities are responsible for taking precautious against
its spread, and apparently the preventive meaaures they
adopt are not very.satisfactory, judging by statistics.
As regards children, in France we only find the boarding
or private school, and there is no', equivalent to our Board
School. If infectious disease should appear in a school
the children attacked are sent home, and notice given to the
parents that they will [not bo allowed to return until the
medical inspector can give them a certificate of health.
The system of inspection of school children regularly every
fortnight by a medical man specially appointed for
the purpose seems a very good one. In the event
of an epidemic breaking out, the medicalj inspector can
request the Mayor to close the school; and this he can do on
his own authority, he being merely required to inform the
administrative authorities of his decision.
In Germany sanitary science has been the subject of much
attention, but this has been chiefly confined to those specially
interested in this matter, and it has not as yet given rise to
any definite Health Acts being formulated by the Govern-
ment. Very precise and careful regulations have been drawn
up, but they lack one mcst essential point; they are abso>
lutely unclassified, and hence when it is necessary to find
out the regulations of any special point, the labour of
searching for them is endless. The details are not very
generally known.
Within certain reasonable limits each State can make itB
own sanitary regulations, the carrying out of which is, aa is
the case in France, in the hands of the police, who appear to
combine the qualifications and duties of both medical officer
and inspector, to some extent^at least. Evidently, however,
sanitation is more intelligently understood judging by the
statistics, which show a steadily diminishing mortality.
In Germany the Chancellor of the Exchequer adds the
supreme direction of Public Health to his other duties, and
he has under him a consulting council, which does the work.
Each province has a President who is referred to in all
matters of health, and the province is divided into districts
and sub-districts, with a duly qualified medical man at the
head of sanitary matters. In addition to the ordinary duties
of inspection and prevention of the Bpread of infectious
diseases, this official has to instruct the police as to how
" each disease spreads and the early symptoms of each."
In Germany the notification of infectious diseases is com-
pulsory, and the head of a family, hotel-keeper, clergyman,
or doctor has to inform the police on the appearance of any
infectious disease, which they think likely to endanger public
health. The police have to see that any such case is ex-
amined by a doctor, and if pronounced of a dangerous
character the police have to inform the administrative
authority of the town or district and the military authorities.
The police have to keep a complete register of all personB
suffering from infectious diseases, and also a daily report of
all such cases to the head of the government of the province.
The diseases that have to be notified are cholera, small-pox,
typhus, typhoid, measles, scarlet fever, diphtheria, and puer-
peral fever.
When infectious disease breaks out in a houBe the doctor
has to decide whether the patient shall be nursed at home
or removed to a hospital, though except in special caseB he
cannot be removed without the consent of the head of the
family. In the event of the patient being nursed at home,
careful regulations are laid down regarding isolation. If the
form of disease is a serious one, the police may cut off the
house from all communication with neighbours. However, if
isolation cannot be properly carried out the police are bound
to hang a black tablet on the house with the nature of the
disease inscribed on it. Such tablet must not be removed
till permitted by the doctor.
No children suffering from any of the notifiable diseases
already named, nor in addition from scabies, contagious
ophthalmia, or whooping cough are allowed to attend school,
nor may any member of the family unless the doctor gives a
certificate. If infectious disease breaks out in a boarding
school, the patient is isolated or removed to an infeotious
hospital, and none of the pupils are allowed to return home
till certified by the doctor as safe.
The regulations for Berlin itself closely resemble those of
the rest of Germany in their administration ; the sanitary
regulations themselves follow those of London very closely.
It possesses an excellent hospital for infectious diseases
called the "Moabit" hospital, where isolation is very
thoroughly carried out.
xxxiv THE HOSPITAL NURSING SUPPLEMENT, April 22, 1893,
IRurstng In tbe Xanfc of ?pbtr.
[By our Special Correspondent.)
HOSPITAL EXPERIENCES AT UMTALI, MANICA-
LAND.
We have had a curious and unique experience here lately,
which may perhaps amuse some of The Hospital readers.
To our great regret, Dr. Johnston, the most delightful person
to work with, was summoned home. His proposed locum, Dr.
Wilson, had gone down to the coast, and thohgh repeated
letters had been sent after him, the day of Dr. Johnston's
departure arrived, without our having been able to obtain
any news of Dr. Wilson beyond a vague rumour that he had
been hunting lions, and was very ill " somewhere." We had
only two convalescents in hospital, and the health of the
district was fairly satisfactory, so, as Dr. Johnston was ob-
liged to go, the opportunity seemed favourable.
He and his dispenser therefore departed, leaving Sister
Lucy and myself to represent nurses, dispensers, district
Burgeons, medical officers, and veterinary t-urgeons for
Manicaland, a tract of country bigger than France, but,
thank heaven, not so populous.
We'gotridof our convalescents in a couple of days, and
had just put the hospital through its usual course of carbo-
lising, when we saw from afar a sight that made our hearts
sink.
A procession was winding its way across the valley. A
native came first, carrying a note stuck in a split reed ; the
aun caught the white paper as he held the reed up before him,
like an acolyte bearing a tall taper ; behind him) came a
machila, or hammock made of canvas, slung on a large, long
bamboo, and borne by four natives. Out of this drooped a
limp-looking leg. Behind the machila walked two extra
boys, but none of them carried any luggage.
We watched that procession through our field-glasses, and
concladed that here at last was the terrible smash we had so
often talked of and dreaded. The machila made Btraight for
the hospital, which is recognisable by its Red Cross fiag.
With the calmness of despair we met it at the gate, and
found it contained a young Englishman who had been taken
ill at Masae-Kesse, where the Mozambique Company have,
unfortunately, no doctor, so the sufferer was sent over to ua.
A journey of twenty miles over hill and dale in a broiling
aun had not improved the condition of a bad case ofjre-
mittent fever. Dr. Johnson had left us an excellent prescrip-
tion for this malarial fever. We had frequently seen it act
like magic, but in this case it proved of no avail. Get that
youth's temperature below 104 deg. we could not. He was
unable to retain'any medicine, and could take no nourish-
ment but a spoonful or two of "Brand's essence" or cham-
pagne given alternately. Decidedly things were notjlooking
well.
We resolved to leave drugs alone and try a wet pack.
Sponging and wet packs generally prove delightfully success-
ful remedies. Already the unhappy patient had tried various
drugs at Masse-Kesse. In one morning he had taken aperient
pills, quinine, sulphate of magncBia, and poached eggs and
beef tea in addition. In Manicaland it is considered exceed-
ingly dangerous to wash sick persons. The Dutch have
introduced this prejudice into South Africa.
However, having to rely on our own judgment, we wet-
packed this patient every four hours, of course taking all
possible precautions, and we had the satisfaction of seeing
the temperature go steadily down. The vomiting also ceased,
and the patient became slowly and i surely convalescent
without a single relapse.
The anxiety of this time was relieved by various amusing
incidents. For instance, a letter came from a farmer at
Borne distance. " The Sisters are requested to send six
sleeping draughts at once by bearer. The writer is very weak
and can't sleep." We had to explain that the administration
of six sleeping draughts might probably be followed by an.
inquest; also that we were not licensed to kill ! It seemed
a case for a mild sedative, which we accordingly sent.
Another patient wrote to say he wanted a purgative
would we send " Calls-cum-colls pills ! " or else some " Hydrg
perchlor powder " would do for him.
Everyone here has a smattering of medical knowledge,
which is truly a dangerous thing. A poor fellow out near
Masse-Kesse insisted on taking some sort of quack medicine
in which'he believed, became rapidly worse, and died as he
was being carried over to us in a machila.
Naturally, after several years of hospital work, we know
something of the [usual treatment of simple cases and the
effect of the more ordinary drugs, and are able to form a
tolerably accurate opinion of what is the matter. Bat, above
all, we have acquired sufficient knowledge to cherish an
extreme dislike to amateur prescriptions, so we carefully
followed in the lines of Dr. Johnston's treatment, and rather
enjoyed the importance of driving 'the donkeys on what we
called our " rounds " and looking after ailments too slight to
be admitted to hospital.
We did not get through our time of responsibility without
taking in an accident:.
A native who had been playing with a detonator came with
half one hand and the tops of the fingers of his other hand
blown off. Accidents of this sort are far less difficult to treat
than complicated medical cases, especially after our experi-
ence at Kimberley, where mishaps of all kinds abounded,
and the dressings were chiefly done by the nurses. So we
got our native's hands soon healed up satisfactorily.
The camp horses took this opportunity of developing bad
eyes and ulcers ; also one of them unluckily staked itself in
a game-pit. We had several sick dogs on our hands, too,
and for a whole day the monkey gave us cause for grave
anxiety.
So far we had a successful medical reign, but, alas ! late
one evening another machila appeared, and a man in a.
state of extreme prostration was brought to the hospital.
Examination proved him to be in a truly terrible condition,
and after lingering for ten days he died in spite of all our
efforts to save him. We concluded that he died of " gumma,"
and signed a certificate to that effect. Of course, we were
very sorry to lose a case whilst there was no doctor here,
but after all the man would have been worse off far away in
Borne lonely hut.
The morning of his death was made memorable to us by an
attack of lions. They suddenly emerged from the bush,
and chased our cattle into the police lines, which are close to
the hospital. This happened at nine a.m., and that same
afternoon, in spite of having been hotly pursued ia tho morn-
ing, the lions reappeared, and hunted the police horses, feed-
ing between our encampment and the township.
The patient's remains were taken to the burial ground in a
cart drawn by oxen, followed by men armed with rifles, all on
the look-out for uncomfortable possibilities. Happily, how-
ever, nothing alarming took place.
The next day Dr. Wilson arrived. He was seriously ill
for many days, but fortunately recovered ; and, after six
weeks of mingled successes and failures, we very gladly
settled into our usual routine and resumed our nursing duties-
GOOD NURSING IN DUBLIN.
The Loxd Lieutenant of Ireland dined with Dr. Kidd,
President of the Academy of Medicine, at the Dublin College
of Surgeons the other day. His Excellency spoke most highly
of the admirable nursing arrangements in the leading hos-
pitals in Dublin, several of which he had visited.
Apeil 22, 1893. THE H0SP17AL NURSING SUPPLEMENT. xxxv
Xtfting patients.
The question of lifting helpless patients is a very difficult
cne. On the one hand a trained nurse is expected to be able
to raiBe up a helpless private patient when he has slipped
down in bed, or when a convalescent is able to make the first
move from bed to a chair or couch, the nurse is expected to
lift him. Some nurses have a wonderful knack in lifting
easily, it seems to come naturally to them, and apparently
they have taken no special pains to learn it. Others again
never seem able to acquire it, however hard they try. Nor
does the power necessarily depend on mere physique, for
although women who are tall and strong and long-limbed
have a natural advantage, still one of the cleverest nurses in
this line the present writer ever knew was a short, rather
stout nurse, whose skill was remarkable.
Our sympathies are entirely with the nurse who is not able
to lift patients easily without the risk of considerable strain,
and we think her case is often a very hard one. There is no
doubt that the public who employ private nurses are often
most unreasonable. If they do not declare it actually in so
many words, their conduct shows only too plainly that,
"having gone to the expense of a nurse,'' they feel they
have a right to get all they can out of her. Talk of hospitals
sweating nurses, indeed! The worst sweaters of all are
found amongst those who employ private nurses, and claim
their pound of flesh to the last ounce. No one resents eo
much the modest request of the nurse for a little help in
lifting a patient as does the employer of the private nurse.
"What! nurse not able to lift B ! What on earth is the
use of her, and just think how much we have to pay for her,
too ! " is the usual exclamation.
There is no doubt about it; some nurses cannot lift
patients, and such should on no account attempt it, for the
results are alike disastrous to patient and nurse. In all
probability the patient will be hauled up.in a manner which
is anything but agreeable, and may even prove hurtful,
whilst the exertion may cause the nurse severe strain and
permanent injury. Moreover, to add to the difficulties of
the private nurse, the beds are frequently most unsuited to
a patient, being high and very wide, even if a feather bed is
not included.
Surely it would not be much to'ask'of the friends that one
person should acquire the little knowledge needed to help tha
nurse to lift her patient, which is the easiest thing possible
for two persons who will work together, and causes no strain
to either, whilst the patient is decidedly the gainer by the
arrangement. All that is,required is that a third person should
volunteer to be the victim, and lying down on a bed should
make herself as helpless and as like a log as possible and
allow the novice, under the nurse's directions, and with her
help, to raise her up and down and turn her from side to side.
If this be practised once or twice tha experiment can be
safely tried on the patient, who, it must be remembered, will
always prove more difficult to move than an active, healthy
person, his very helplessness making him a dead weight.
Besides, an inexperienced person has often a quite needless
fear of hurting a patient, and for that very reason will cause
pain. Thus the periodical move will no longer bo a moment
dread alike by patient and nurse. One word of warning is
necessary, that the nurse should entirely discourage any
silly chatter over the patient during bed making. Few things
are more irritating than such foolish talk as "Now, up with
him," " That's right " in a tone of voice like that in which
some people talk to children, and intended, we believe, to be
reassuring, but in reality most irritating. The various steps
of sheet changing should be carried out in a regular order,
familiar alike to nurse and assistant, and aB little talk
indulged in as possible the while.
Everpbobg's ?pinion.
[Correspondence on all subjects is invited, but ice cannot in any vm%
be responsible for the opinions expressed by our correspondents. No
communications can be entertained if the name and address of tht
correspondent is not given, or unless one side of the paper only ba
written on J
ATTAINABLE PERFECTION.
"A Private Nubse" writes: In reference to the article
under the above heading, which appears in the "Nursing
Mirror " of April 1st, allow me to say how thoroughly I agree
with it, particularly that part which urges the necessity for
special training for private nurses. It seems to be a generally
received opinion that because a woman haB spent two or three
years at hospital work, and proved herself efficient there, she
is fitted to go out as a private nurse. There could be no
greater mistake. The class of patients we meet in general
hospitals is, as a rule, entirely different to that with which
we come in contact in their own homes, and the very
qualifications which make a nurse suitable for work
amongst one class often unfit her for dealing with the other.
It would be a good thing if all probationers had clearly
before their minds what form of nursing they intended to
follow on the expiration of their training, and if private work
should be the object of their ambition, let them qualify
properly for it by a short period passed in a Homo for paying
patients of the better class. In this way they will gain some,
insight into what will be expected of them, though of the
many trials from disagreeable surroundings, officious friends,
and other trials that beset the path of the private nurse they
may still be ignorant. As things are, we practise on our
patients and their households with frequently disastrous
results, buying our experience at their expense.
HOW NOT TO NOURISH BABIES.
Nurse Helen writes : I was very much amused with
your paragraph, "How Not to Nourish Babies." I get
wonderful confessions as to the diets which infants are treated'
to. One child had been brought up, I remember, without
any milk at all. Beef tea, water, arrowroot, and biscuits,
soaked in hot water and sweetened with brown sugar, being;
part of the unfortunate infant'B miscalled "nourishment."
It was difficult to persuade the mother that the child's
delicacy and, later on, its development of "rickets" were
the outcome of her ignorance. When these poor mothers
have learnt wisdom, then only will the babies set duly
nourished.
A CORRECTION.
Sister Katherine writes : Thank you very much for
the notice of St. Mary's Home, Plaistow, inserted in The
Hospital. I feel bound to call your attention to one point.
The thirty nurses are not all trained and certificated ; the
larger number of them are pupils, this being a training;
school. The heads over each department aie fully-trained
nurses. If you can spare space I should be grateful indeed if
you would mention our need for gifts of letters for convalescent
homes for children and also adults. There are always so
many patients for whom we long to secure a change in the
summer months. We are all very grateful for the kind help
already received through The Hospital. We are in great
want of clothes for both children and adult3.
Beatb in our IRanfcs.
Miss Mary Ann Groser died on the 11th inst. of acute
pneumonia after three days' illneBs. Nurse Groser waa
trained at the Nurses' Institute at Exeter, and during the
cholera outbreak in that city in 1865 she was placed in chief
charge of the patients. Three years later she was appointed
to the Corporation Hospital at Plymouth, and there worked
through another small pox epidemic. Afterwards she was
for seven years matron of Penzance Infirmary. For the last
nine years Miss Groser haB worked as parochial nurse in the
mining district of New Benwell, where her skilled attention
was greatly valued by her patients, by whcm her death i&
deeply regretted.
xxxvi THE HOSPITAL NURSING SUPPLEMENT. apeil 22, 1893.
Hbe 3ubilee 3nstttute at Taunton,
The Jubilee Institute at Taunton was ostensibly founded
to provide nursing in their own homes for the Bick poor of the
town and surrounding districts. This object appears to have
been entirely lost sight of as soon as the necessary funds
were collected. The Institute, we are told, has done its work
exclusively in the houses of the well-to-do. This extra-
ordinary condition of things is only now made clear to the
public by the action of the Taunton District Nursing Associa-
tion, which carries out the work amongst the poor left
undone by the Taunton Jubilee Institute. The District
Nursing Association modestly asks for a grant from the funds
(some ?5,000) of the Taunton Jubilee Institute. Now that
the existing state of things has been so efficiently ventilated,
we muBt hope that justice will shortly be done in this matter,
as the suggestions of reparation made by Mr. Cosens at the
annual meeting were cordially approved by those he
addressed.
?be East Xonbon Ibospftal for
CbUDren.
On the 17th inst. His Royal Highness the Duke of
Cambridge presided at the annual festival dinner of the
East London Hospital for Children, which was held in the
Whitehall Rooms at the Hotel Metropole. Amongst the
guests present were the Earl and Countess of Erroll, Lady
Edmund Talbot, Lord and Lady Muncaster, Lord Downe,
Mr. Charles Cheston, and many other friends of the hospital.
Knowing the thorough examination always made by the
Duke into the financial and general management of in-
stitutions to which he gives his support, his tribute
to the accounts on this occasion is most valuable,
for he called them very satisfactory. He referred to
the need for increased support for a useful hospital
situated in such a poor and populous district, and he sug-
gested that disagreeable things, such as begging, could
be rendered almost agreeable if done in a pleasant manner.
Referring to the low condition of the funds at other hospitals,
the Duke genially remarked that he not only hoped for a
liberal response to his appeal on the present occasion, but
also when he had to ask again for help'in other directions.
He begged that the cold weather experienced that day should
not be allowed to affect the charitable feelirigs of his hearers.
The Duke's speech was received with hearty applause,
though no one appeared willing to endorse the too
modest valuation he put on the services which he
has himself rendered all his life to the metro-
politan charities. Mr. Cheston, chairman of the hospital
spoke with great regret of the absence; through ill-health of
the Earl of Strafford, who had done so much for the East
London Children's Hospital. Referring to his Royal
Highness' commendation of the balance-sheet, Mr. Cheston
remarked that he not only liked to see the pennies figuring
well in accounts, but he also thought that every penny given
to a charity should be well accounted for. Mention was
made of Baron Hirsch's handsome gift, and also of a special
donation by Mr. Galpin of ?1,000 towards the convalescent
home. Mr. Cheston thought that the popularity of the Royal
Family was very largely due to their recognition of there
being two sides to London, namely, the east and west.
Lord Erroll, Mr. Vaughan Morgan, Mr. Trinder, and other
gentlemen were amongst the speakers. Such universal
regret was expressed at Mr. Cheston's proposed retirement
that possibly he may still be induced to retain the office of
Chairman to the hospital in which he has proved his interest
most practically. In the course of the evening the Secretary,
Mr. Whitford, announced the receipt of subscriptions to the
amount of ?1,200.
draining in Gbilbren's Ibospitate.
So often do inquiries reach us as to rules for probationers in
children's hospitals that we propose to answer some of these
by giving a short summary of the regulations at the East
London Hospital at Shadwell.
To begin with the important subject of age. Nurse Pro-
bationers are received from 20 to 26, and Lady (or Paying)
Probationers from 20 to 30.
The work in the wards isjthe 'same for both, but the
Paying Probationers do not go on night duty during the first
six months. They have six weeks holiday during the year
and the Nurse Probationers have a month. Three hours are
allowed off duty daily to both, but it is not specified whether
this is exclusive of meal times.
Uniform dresses, aprons, and caps, as well as a reasonable
amount of washing are allowed for the Nurse Probationers.
The Lady Probationers provide all these things for themselves
and pay at the rate of a guinea a week for their board and
training.
For the Nurse Probationers we read " classes for instruc-
tion will beheld when practicable." Also that they are paid
?10 for the first year, ?12 for the second, and ?20 for the
third. All probationers have a day and night's leave of
absence every month, and the Lady Probationers have in
addition from two to ten every Saturday.
The regulations to be observed by Nurses and Probationers
at the East London Children's Hospital are much the same
as those in force at other training schools. The Lady Proba-
tioners enter for six or twelve months and the Nurse Proba-
tioners are bound to the service of the hospital for three
years.
The rules drawn up for the Sisters are also similar to those
of general hospitals. The time allowed them off duty is very
liberal on four days of the week, namely, five to ten Tues-
day, six to ten Thursday, two to ten Satursday, five to ten Sun-
day, bat on the other three days the Sisters appear to befour-
teen hours on duty. They have four weeks' annual holiday and
once every month from two o'clock on Saturday to the
following Monday morning. Indoor uniform, board and
" a reasonable amount of washing," are also provided for the
sisters and a salary of ?30 first year, ?35 second year, and
?40 third year, is given.
flotes anb ?ucrics.
Queries,
(109) Mental Training.?Can anyone tell me where I can obtain
systematic instruction in the theoretical and practical nursing of persons
suffering from mental diseases ??Probationer Nellie.
(110) Stains.?How can I efface stains made on linen by Ferri.
Perohlor ??Nurse Kate.
(111) India.?Kindly advise me where I can got information as to up-
country nursing in India ??Traveller.
(112) Quarantine.?I want to know of any place where nurses are
taken in duriDg quarantine after infection ??Ethel.
(113) Cripples.?Can you tell me the name of institution where arti-
ficial limbs are supplied to the crippled poor ??District Nurse.
(114) India.?I shall be glad to have information about Indian
Nursing Service and Army and Navy nursing generally.?Sister S.
Answers.
(109) Mental Training (Probationer Nellie),?You can get thorough
and complete training at Berrywood. There is special aocommodation
at that asylum for pupil nurses. Write for terms to Medical
Superintendent, Berrywood, Northampton.
(110) Stains (Nurse Kate),?We fear you cannot obliterate them if
they have dried in.
(111) India (Traveller). ?You had better apply to Messrs. Henry King,
Oomhill, who aro receiving subscriptions for the Up-country Nursing
Association.
(112) Quarantine (Ethel).?There is "a Home for Quarantine
Nurses" opened by Mrs. Mence, at 36, St. John's Road, Upper
Holloway.
(113) Cripples (District Nurse),?You will find this information, and
mueh more of special value to nurses, in " Burdett'a Hospital Annual,'
Scientific Press, 428, Strand ; price 5s.
(114) India (Sister S.?See answer to (113).
April 22,1893. THE HOSPITAL NURSING SUPPLEMENT. xxxvii
ZLbe 1R.3S.B.B. in etnntmvgb.
We give below the report of the meeting recently held in
Edinburgh, which pressure on our columns prevented our
inserting last week. It is taken from the Scotsman of 6th
April:?
A meeting of medical men and nurses was held yesterday
afternoon in the Waterloo Rooms, Edinburgh, for the pur-
pose of forming a Scottish branch of this Association. Dr.
Joseph Bell, one of the vice-presidents, occupied the chair,
and amongst others present were Sir Douglas Maclagan,
Professor Grainger Stewart, Dr. Peel Ritchie, Dr. Underhill,
and between forty and fifty ladiea and gentlemen. A tele-
gram was read from the Princess Helena, President of the
Association, to the following effect: "Your meeting has my
warmest wishes. I hope that the work I have so dearly at
heart may be satisfactorily established for the welfare and
good of the nurses in Scotland." On the motion of Professor
Grainger Stewart, seconded by Dr. Peel Ritchie, it was
resolved to send a reply thanking her Royal Highness for her
gracious message. The Chairman made a statement of the
objects of the Association, which, it appeared, was founded
a few years ago, mainly for the purpose of establishing a
register of nurBes, with the view of safeguarding the
hospitals, the public, and the medical profession against
imposition by untrained or otherwise unfit nurses. He was
followed by Dr. W. Bezly Thome and Dr. Fenwick, London,
members of the deputation, who explained more fully
the provisions of the scheme. The former mentioned
that it was exceedingly likely that they would soon be
granted a Royal Charter. The latter spoke of the benevo-
lent side of the aims of the Association. On the motion of
Sir Douglas Maclagan, seconded by Dr. Underhill, it was re-
Bolved to form & branch of the Association for Scotland, with
its headquarters in Edinburgh. Dr. Peel Ritchie put some
questions to the deputation with the view of eliciting what
the Association meant to do to safeguard the interests of
Scottish nurses. Under the scheme no nurse could be regis-
tered unleES she had had three years' training. But many of
the hospitals in Scotland, he pointed out, only exacted one
year's ira'ning as a qualification, and unless something were
done to modify the three years' provision the bulk of
Scottish nurses would be cut off from participation in the
scheme. Dr. Fenwick replied that if a Royal Charter were
granted a period of grace would be given, in which nurses
who had not that qualification but were recommended by
medical meu or matrons of institutions would be allowed to
register. Dr. Peel Ritchie replied that his objection applied
as much to the future aa to the past, as many nurses in Scot-
land could not get a three years' training. He suggested
that one year's training, with two years' connection with a
training institution, should be accepted as a qualification for
registration. Ultimately a motion recommending this pro-
posal to the consideration of the General Council of the Asso-
ciation in London was adopted. Dr. Affleck also spoke, and
urged generally that registration would really be no guarantee
to the public that a nurse on the register was efficient. To
these Dr. Thorne and Dr. Fenwick both replied, lhe meet-
ing, which had lasted about an hour and a half, then finished
by passing a vote of thanks to the chairmaD. Afterwards a
telegram was sent in the chairman's name to the Princess
Helena announcing the meeting's approval of the formation
of a Scottish branch.
IRurses in 1Rew> 3erse\>, 1113,H.
A. New Degree.
The New Jersey State Legislature has certainly proved its
own intention to make nursing into an acknowledged " pro-
fession.^ A Bill has recently been passed to the effect " that
any training school organised, or to be organised, under the
Act to which this is a supplement, or under any supplement
thereto, may confer the degree of Medical and Surgical Nurse
upon any of its graduates, under Buch rules and regulations
as such training schools may prescribe." What the degree is
to consist in, and what the precise rank to which such nurses
will attain, are questions of interest. Whether probationers
are to emerge into nurses with " degrees" at the end of one,
two, or three years' training, is a point oe which there should
be some unanimity, we presume, in the training schools
themselves?
for IReabing to the Sick.
THE PRESCRIPTION.?I.
On asking a young woman the other day whether there was
no remedy for the severe pain from which she was suffering,
she replied, "Oh ! yes, the doctor has written me ajprescrip-
tion, and I mean to have it made up and take it some day."
" Why not 'at once," was the rejoinder ; "it may be too
late when you) fly to the medicine ; " but she seemed quite
content to know there was a cure close at hand, and to go
on as she was, " for it would be such a trouble to take it
regularly," she said ; and besides, she added, " I have not
time to get it."
"But the neglect will probably cause your death," con-
tinued the friend. " No, it will not come to that," was the
answer ; "I shall see to it all in good time." And so she
went on, heedless of consequences, like a child playing on
the brink of a precipice.
Many and many a one acts in this way for the sickness of
the soul, and delays to fly to the prescriptions our Great
Physician has given us in the Bible. There is no ill for which
we may not find relief if we only exert ourselves to get them
"made up." Suffering, sorrow, anxieties, petty annoyances,
can each and all find their antidote in this wondrous book,
while for the heart3 burdened by the guilt of sin, longing to be
quit of the iniquities which weigh it down, there is God's great
prescription, "The Blood of Jesus Christ His Son cleanseth
from all sin." In His Precious Blood we can wash and be
clean, only we must go to the Bath ourselves. The means of
cure is written out for our use just aB was the one given to
the young woman, but if we only look at it and say, I know
it is the right thing, is it really necessary to take it at once ?
I shall wait till a more convenient Beaaon, I think; what
advantage is it to us ? Alas ! for us if we tarry by the way.
Let us hasten, while we have time, to the Fountain which is
open for all, and seek the great Healer who looks on us
with sympathy and love, even when we keep aloof, because
He knows what it was while on earth to suffer hunger, and
thirst, and fatigue, and the agony of dying. And knowing,
He holds out this precious medicine, this " cure all," even
Himself by whom our bodies and souls shall be preserved
unto everlasting life.
But there must be no delay, for now is the appointed time,
now is our opportunity. Remember the Holy Ghost saith
" To-day.''
The Great Physiuian now is near,
The sympathising Jesus.
He speaks the drooping heart to cheer,
Oh ! hear the Voice of Jesus.
appointments.
[It is requested that successful candidates will send a copy of their
applications and testimonials, with date of election; to The Editor,
The Lodge, Porchester Square, W.]
Chelsea HosriTAL for Women.?Miss Ada Plumb has
been appointed Matron of this Hospital.
Middlesborough Sanatorium.?Miss Catherine Bell has
been appointed Matron of the Middleborough Sanatorium.
Miss Bell was trained at the Monsall Hospital, Manchester,
where she afterwards held the post of night superintendent.
We wish her every success in her new sphere of work.
fflMnor appointments.
Nurses' Co-operation.?Miss Jessie Pick has been ap-
pointed Home Sister at the Nurses' Co-operation, 8, New
Cavendish Street. Miss Pick was trained at the Women's
Hospital, Soho Square, and has since held several posts
which specially fit her for her new work.
xxxviii THE HOSPITAL NURSING SUPPLEMENT. April 22.1893.
1ber better Self.
I.?Taking Flight.
" You won't forget me, Daphy ? Promise ! " said '.he, in a
forlorn voice.
" As if I could, Harry ! Never, never !'' said she, throw-
ing aa much energy as she possessed into^the answer.
The two were standing in the lane which by-and-bye
would be white and perfumy with May, but at the moment
was ankle-deep in the slush of February. Little cared the
young lovers, however, for the shrill, biting wind that
whistled through the branches and swirled last summer's
dead leaves up into their faces. To Harry Dunsford the
draughty, damp lane was simply Arcadia itself; and for the
girl by his side, her eyes were fixed,vaguely on the distance,
through the hedge-gaps, on an Arcadia^of her own?that
promised land of streets and shops, of pomps and vanities, of
joys and pleasures, we call London Town.
Daphne Reece, her father's idol, and che youngest of all
his children, was the only one left in the home of his old age.
Like all over-petted things, she had been allowed to grow
up anyhow. But it did not seem to have done Daffydown-
dilly, as her father loved to call her, much harm, not out-
wardly at least, for she was sweet and fair as a girl should
be. Of course such a comely maiden had a lover; it was
natural as flowers in May that it should be so. Young
Harry Dansford, the gentleman farmer, who had come to
settle in the neighbourhood, speedily "gave his heart away,
and did his best to win that of Daphne in exchange. But
the girl was shy, wayward, and, at times, wilful to distrac-
tion ; so Harry, after the manner of most lovers,'Jived iD a
troubled sea of doubts, and fears, and ecstatic surprises.
There came about one surprise, however, which was any-
thing but ecstatic.
"I've got such news !" Daphne had breathlessly told the
young man one Sunday when Harry caught her up just out-
side the churchyard in order to accompany her home.
So elated was the girl that she totally forgot to exhibit the
pretty pantomime of mock astonishment and doubts as to the
propriety of the thing, which was demurely gone through
Sunday after Sunday, when Harry unfailingly Joffered his
services to escort her to The Bunk, as the oldjaaval officer,
her father, called his comfortable riverside cottage.
"What's your news, Daphy?" aeked Harry, absently.
His eyes and his thoughts were too fully occupied with the
fair human picture by his side to feel any great interest in
such a aublunary thing as news. Besides, the present was
all-perfect ; there was no need for anything beyond.
" Father had a letter last night from London, from Aunt
Sarah, and I am to go up to her for the season to "
Daphne faltered abruptly, her dimples coming and going for
some reason.
Harry, on his part, had pulled up short; there was a dull
surging in his ears, and the sky, which had been strangely
blue for February in his eyes the moment before, grew
blurred and grey; the world stood still; but he neither spoke
nor moved. Thus it is we train ourselves to meet the various
shocks that spring upon us in this life of ours. The mouth
takes a grim set, and the figure braces itself into straight-
ness; those are the outward and visible signs that an
Englishman has received a heart-hurt.
"You don't seem to think my news very interesting !"
Daphne affected to be aggrieved, but she studioully avoided
looking at Harry's face.
" What does this?this Aunt Sarah want with you,
Daphy ? " he asked at laet, and for a moment his voice fairly
startled the girl, it was so harsh, so altered.
"She says, in order to give me a chance !" Out came all
the dimples in full play.
" I thought so !" groaned Harry, "I knew it!" Then
there were a few words of that nature which the Arabs
declare come home to roost. At any rate, the expressions
were not quite complimentary to Aunt Sarah, and would
undoubtedly have shocked that exemplary lady. " I beg
your pardon ! " said Harry feverishly, " but, oh my littlo
darling, I know how it will end. They will separate us?
part you and me. If they do, I'll?"
"You'll find somebody better!" calmly put in Daphne,
Intercepting the alarming threat. " How absurd you are !
Why shouldn't I go and see the world and?and have my
chance as it's called ? Why shouldn't 1 as well as any other
Daffy downdilly
' go up to town
In a green petticoat and yellow gown "I"
The girl looked up bawitchingly as she hummed the
words, and Harry writhed in helpless fury. He had not as
yet been able to obtain Daphne's consent to a formal engage-
ments She wouldn't hear of it. Now, the result would be
that she would slip out of his fingers, he was dismally sure
of it. Even when the parting came, a few days later, all he
could extorb from the wayward girl was a promise not to'
forget him, nothing more.
And so Daffydowndilly went up to town.
Daphne Reece had been over a month in town. A little
of the strangeness of,the wonderful new life was beginning to
wear away, but not yet could her ?yes see true because of its-
glamour.
Aunt Sarah was an admirable hostess ; her household a?
well-conducted one, and she had launched Daphne into tho
swim of the season.
"I make a point of always having a girl to take about,'''
said she complacently ; "I call them my excuses. If they
do well?you understand, my dear," the old lady's head nod-
ded significantly, "I call them my rewards. But if they are
failures, I call them my accusations ! For the present you
are my excuse,Daphne ; don't, pray, turn into an accusation,
you're much too good looking for that fate."
So, to the girl's unqualified delight, Daphne was taken a
racketting round of ball and play and dinners, and the far
away riverside home, the indulgent old father, and the faith-
ful lover grew smaller [and fainter to her mind's eye until!
they each became well-nigh blotted out. It was as though
she looked at the past, near though it was, through the
wrong end of a telescope ; the present belittled it; and she
grew to wonder disdainfully at her old content.
As for Harry, she blushed in her own face, before
the glass, to remember that she had ever thought of him
as a possible lover. How thankful she felt that she was still
free. Harry, indeed ! A queer figure he would make among:
the fashionable smart folk she, now, believed constituted the
world. Of her lover's sterling worth, his upright, brave
character ; the tenderness of his large nature?the true gold?
foolish Daffydowndilly remembered just nothing at all. Her
sweet eyes were too much dazzled by the glitter of the sham
metal.
"You like the life?" asked Aunt Sarah drily as she
regarded the eager, beautiful face of her excuse. " Well, it
will be your fault, if you don't make it your own. You've
got your chanse ; anybody can see you've only to hold up-
your little finger "
" Don't, Aunt Sarah ! " Daphne's fair face flamed crimson,
and her heart beat in a wild hurry. For the first time she
realised her position, half-promised to one lover, and warding
off a proposal from another, one of Aunt Sarah's choosing*
eligible at all points, one might be sure.
(To be continued.)
Wants an& Workers.
Wanted, a Convalescent Home wliere a young man with open wonnS
could be receivtd for six or eight weeks. Stat3 terms, including dress-
ings for wound (amputation of leg).?H.A.R,
April 22, 1893. THE HOSPITAL NURSING SUPPLEMENT. xxxix
She Book TCIorib for Women an& IRurses.
[We incite Correspondence, Criticism, Enquiries, and Note? on Books likely to interest "Women and Nurses. Address, Editor, The Hospital
(Nurses' Book World), 428, Strand, W.O.l
Island Nights' Entertainmwvt t> t
aai^HD 1N1UHTS ENTERTAINMENT. By R. L. SlEVHNSON.
(Cassell and Co.)
?? Good wine needs no bush," and good books need no critics.
When the whole of a large first edition is disposed of before
publication, as is the case with the " Island Nights' Enter-
tainments," the public evidently do not require much
exhortation on its merits. Yet it is perhaps safe to prophesy
that some readers will be disposed to turn away from their
"Entertainment" when they perceive that the islands are
in the far Pacific, that the men and women of whom it treats
are "Kanakas," and that much of the conversation is
?carried on in a kind of pigeon English, less irritating perhaps
than actual phonetically-spelt dialect, yet presenting fewer
possibilities of expression. But once fairly landed on the
Beach of Falesa, all these apparent stumbling-blocks are seen
to bs new features of beauty. The story marches with all
the Bwinging tread and precision Mr. Stevenson has drilled
into his creations, and before the first dozen pages are over
the reader is in the thick of the situation, and as deeply
absorbsd in the affairs of Falesa and the prospects of its new
white trader as though he had spent his life on thejjisland.
It is the trader, Wiltshire, who tells the story. Here is his
description, on the first night of his arrival, of his mock
marriage with the Kanaka girl Uma, who may be called
Mr. Stevenson's first real heroine.
"The sun was down, the Bky all on fire, and the lamp
had baen some time lighted, when Case came back with Uma
and the negro. She was dressed and scented ; her kilt was
of fine tapa, looking richer in the folds than any silk ; her
bust, which was of the colour of dark honey, she wore bare,
only for some half-a-dozen necklaces of seeds and flowers ;
and behind her ears and in her hair she had the scarlet flowers
of the hibiscus. She Bhowed the best bearing for a bride
conceivable, serious and still; and I thought shame to stand
up with her in that mean house and before that grinning
negro. I thought shame, I say ; for the mountebank was
dressed with a big paper collar, the book he made believe to
read from was an odd volume of a novel, and the words of
his service not fit to be set down. My conscience smote me
when we joined hands ; and when she got her certificate I
was tempted to throw up the bargain and confess."
The subsequent courtship is very charming. It is not long,
however, before Wiltshire discovers that he has been tricked
by the man Case into marrying a girl who is under " taboo,"
and that none of the natives will have any dealings 'with
him. It ia Uma herself who tells him. " I 'shamed," she
said ; " I think you savvy. Case he tell me you savvy ; he
tell me you no mini; tell me you love me too much. Taboo
belong me," she said, touching herself on the bosom as she
had done upon our wedding night. " Now I go 'way ; taboo
he go 'way too. Then you get too much copra. You like
more better I think. Tofa, alii," says she in the native :
" farewell, chief." " Uma," I said, " I would
rather have you than all the copra in the South Seas."
The mission boat reaches the island opportunely the same
afternoon. "She was a long whaleboat, painted white; a
xl THE HOSPITAL NURSING SUPPLEMENT. Apkil 22,1893.
THE BOOK WORLD FOE. WOMEN AND NUE>SES?continued.
bit of an awning astern, a native pastor crouched on the
edge of the poop, steering; some fourand-twenty paddles
flashing and dipping true to the boat song; and the mis-
sionary under the awning, in his white clothes, reading in
a book, and sipping tea ! It was pretty to see and hear;
there's no smarter sight in the islands than a missionary
boat with a good crew and a good pipe to them
We walked right in through the store, and I was sur-
prised to And Uma had cleared away the dinner things.
This was so unlike her ways that I saw Bhe had
done it out of gratitude, and liked her the better. She and
Mr. Tarleton called each other by name and he was very
civil to her seemingly. ' Uma,' said I, ' give us your
marriage certificate.' She looked put out. ' Come,' said
I, 'you can trust me, hand it up.' She had it about her
person as usual; I believe she thought it was a pass to
heaven and if she died without having it handy she would
go to hell. ' Now,' said I, with the certificate in my hand,' ' I
was married to this girl by Black Jack the negro. The
certificate was wrote by Case, and it's a dandy piece of
literature I promise you. Since then I've found that there's
a kind of cry in the place against this wife of mine, and as
long as I keep her I cannot trade. Now what would any
man do In my place, if he was a man? I said. 'The first
thing he would do is this, I guess.' And I took and tore
up the certificate and bunged the pieces on the fioor.
"' Ave !' (alas), cried Uma, and began to clap her hands,
but I eaught one of them in mine.
" ' And the second thing he would do,'' said I, " if he was
what I would call a man, and you would call a man,
Mr. Tarleton, is to bring the girl right before you or any
other missionary, and to up and say, "I was wrong married
to thia wife of mine, but I think a heap of her, and now i
want to be married to her right." Fire away Mr. Tarleton.
And I guess you'd better do it in native ; it'll please the
old lady,' I said, giving her the proper name of a man's
wife on the spot. So we had in two of the crew for to
witness, and were spliced in our own house ; and the parson
prayed a good bit, I must say, but not so long as some, and
shook hands with the pair of us." The Kanaka bride is, in
fact, as fascinating a person as can be met in a day's journey
through a library.
PERIODICALS.
Cornhill Magazine.?This magazine for April is specially
rich in specimens of good fiction. Baring Gould and W. E.
Norris both contribute serials. There are also interesting
articles on "Actors and Actresses in Westminster Abbey "
and " Our Arctic HeroeB." Lovers of the charming Hamp-
shire coast find the adjacent country will be pleased with an
article entitled *' Christchurch Bay," which is full of life
and interest.
Cassell's Family Magazine.?If Messrs. Cassell main-
tain the high standard to which they have now brought their
magazine, we prophesy that it will speedily grow in popu-
larity. Both illustrations and contents for this month are
excellent, and almost every portion of the community will
find some subject of interest ia its pages, and all for the
modest price of sevenpence.
St. James' Budget.?We have a specimen of this weekly
illustrated paper before us, and are much struck with the
large number and excellence of the illustrations. Thia
number will be of special interest to many, as it contains an
interesting article on, with some really artistic reproductions
of, the Spitzer Collection, which most of us are curious to
see, and which will so soon be scattered all over thg world.

				

